# Evidence for Intramolecular Antiparallel Beta-Sheet Structure in Alpha-Synuclein Fibrils from a Combination of Two-Dimensional Infrared Spectroscopy and Atomic Force Microscopy

**DOI:** 10.1038/srep41051

**Published:** 2017-01-23

**Authors:** Steven J. Roeters, Aditya Iyer, Galja Pletikapić, Vladimir Kogan, Vinod Subramaniam, Sander Woutersen

**Affiliations:** 1 Van ’t Hoff Institute for Molecular Sciences, University of Amsterdam, Science Park 904, 1098 XH Amsterdam, The Netherlands; 2 Nanoscale Biophysics Group, FOM Institute AMOLF, Science Park 104, 1098 XG Amsterdam, The Netherlands; 3 Dannalab BV, Wethouder Beversstraat 185, 7543 BK Enschede, The Netherlands; 4 Vrije Universiteit Amsterdam, De Boelelaan 1105, 1081 HV Amsterdam, The Netherlands

## Abstract

The aggregation of the intrinsically disordered protein alpha-synuclein (*α*S) into amyloid fibrils is thought to play a central role in the pathology of Parkinson’s disease. Using a combination of techniques (AFM, UV-CD, XRD, and amide-I 1D- and 2D-IR spectroscopy) we show that the structure of *α*S fibrils varies as a function of ionic strength: fibrils aggregated in low ionic-strength buffers ([NaCl] ≤ 25 mM) have a significantly different structure than fibrils grown in higher ionic-strength buffers. The observations for fibrils aggregated in low-salt buffers are consistent with an extended conformation of *α*S molecules, forming hydrogen-bonded intermolecular *β*-sheets that are loosely packed in a parallel fashion. For fibrils aggregated in high-salt buffers (including those prepared in buffers with a physiological salt concentration) the measurements are consistent with *α*S molecules in a more tightly-packed, antiparallel intramolecular conformation, and suggest a structure characterized by two twisting stacks of approximately five hydrogen-bonded intermolecular *β*-sheets each. We find evidence that the high-frequency peak in the amide-I spectrum of *α*S fibrils involves a normal mode that differs fundamentally from the canonical high-frequency antiparallel *β*-sheet mode. The high sensitivity of the fibril structure to the ionic strength might form the basis of differences in *α*S-related pathologies.

The formation of amyloid fibrils (characterized by a so-called cross-*β* structure^1^,^2^ that is stabilized by an intra- and intermolecular hydrogen-bonding network) is currently known to be related to approximately fifty disorders, including Alzheimer’s and Parkinson’s disease, and type-II diabetes^3^. In the case of Parkinson’s disease, amyloid aggregates of alpha-synuclein (*α*S) are found in the Lewy bodies, that are an import hallmark for the disease^4^,^5^. Although physiochemical conditions that influence the conversion of monomeric *α*S to amyloid fibrils have been investigated before^6^,^7^,^8^,^9^,^10^,^11^,^12^, the structural characterization of *α*S amyloid fibrils is yet incomplete. Elucidation of the molecular details of the *α*S fibril structure is essential to understanding the mechanism of self-assembly of *α*S into fibrils, which is thought to play a role in the pathogenesis of PD. It is known that both the conformation of monomeric *α*S^6^ and the structure of its amyloids^12^ depend strongly on the ionic strength of the buffer solution. This is probably related to the long-range interactions within the protein^13^,^14^,^15^,^16^,^17^ that are a result of the charges present in *α*S: at neutral pH the C-terminus (residues 100–140) is highly negatively charged (−13), whereas the rest of the protein has a net charge of +4. To investigate how ionic strength influences *α*S-fibril structure and the conformation of the monomeric subunits within the fibrils, we have studied the aggregation of the full-length *α*S protein at different salt concentrations using a combination of atomic force microscopy (AFM), circular dichroism (UV-CD), X-ray diffraction (XRD) and 1D- and 2D-infrared spectroscopy (1D-IR and 2D-IR).

## Previous ss-NMR studies: *α*S fibrils are “in-register”

There is considerable evidence from solid-state NMR^7^,^10^,^18^,^19^,^20^,^21^,^22^,^23^ and EPR^24^,^25^ suggesting that *α*S fibrils independent of the ionic strength of the buffer solution in which they are aggregated, have in-register monomers along the fibril axis (*i.e.* the residues of one monomer are next to the same residues in the neighboring monomers in the hydrogen-bonded fibrillar intermolecular *β*-sheet; see Methods section for a detailed description of the nomenclature used to describe the fibril morphology). Although fibrils aggregated in high- and low-salt buffer solutions both are in-register, the structure of the fibrils does change dramatically if the protein is aggregated in a low-ionic strength buffer solution (5 mM Tris-HCl), compared to aggregation in a high-ionic strength buffer solution (50 mM Tris-HCl and 150 mM KCl)^7^,^10^.

## Previous FTIR studies on the structure of *α*S fibrils

Vibrational spectroscopy in the amide-I region (1600–1700 cm^−1^) can be used to gain more insight into the conformation of *α*S molecules within the fibril. Due to the strong vibrational coupling between the amide groups in backbones of proteins, amide-I IR spectra exhibit distinct features for different secondary and quaternary structures^26^,^27^,^28^,^29^,^30^,^31^,^32^. Many groups have used conventional IR techniques (the KBr pellet method, solution FTIR or ATR-FTIR) to investigate the structure of *α*S fibrils aggregated under different conditions. The *β*-sheet structure in fibrils prepared in 10 mM HEPES without any additional salt was reported to be fully parallel^33^, but it is still unclear which type of *β*-sheet structure occurs in fibrils prepared at higher salt concentration: many reports assign their IR spectra of high-salt fibrils (aggregated with 137–200 mM NaCl present in the buffer) to an antiparallel *β*-sheet structure^34^,^35^,^36^,^37^,^38^,^39^, but other studies^7^,^40^ report spectral assignments to fully parallel *β*-sheets for *α*S fibrils aggregated in 50 mM Tris-HCl and 150 mM KCl-, and in PBS-buffer, respectively.

## 2D-IR on amyloid fibrils

2D-IR spectra are less ambiguous in the assignment of secondary structure than 1D-IR spectra, as they contain a secondary-structure dependent cross peak pattern, and because highly ordered secondary-structure elements give stronger signals than less-ordered structures^32^. The comparison of the 1D-IR spectrum (in which the total oscillator strength is to a good approximation conserved) and the 2D-IR spectrum (in which the signal is nonlinearly dependent on the transition dipole of the IR modes^41^,^42^) provides a measure of the excitonic delocalization of each structural component, making a combination of the techniques exceptionally useful to study amyloid systems, as has been shown by the work of the Zanni group^43^,^44^,^45^,^46^.

## Results

### Comparison with other studies using conventional techniques

**Figure 1:**
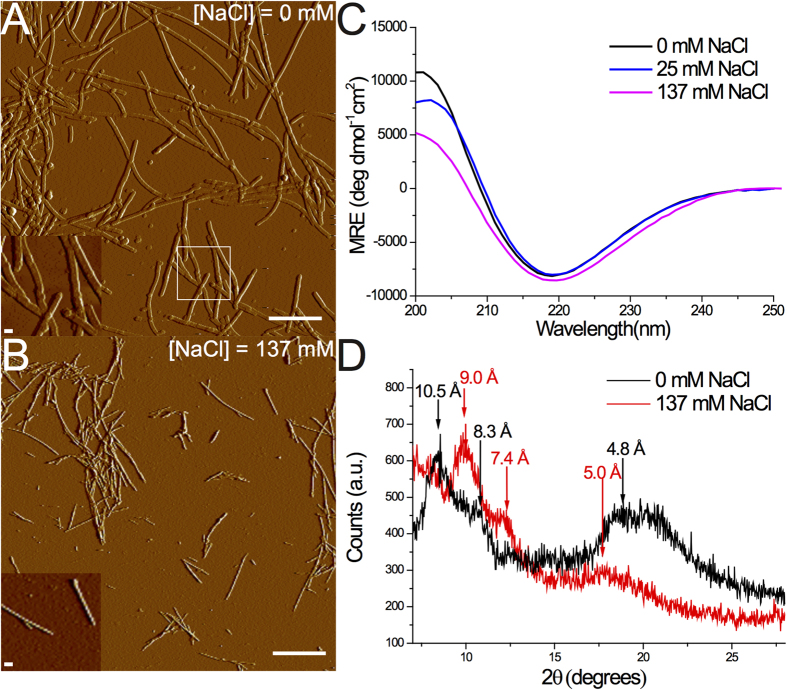
(**A**,**B**) AFM images (scale bar = 10 *μ*m in main image and 100 nm in the inset), (**C**) UV-CD spectra, and (**D**) XRD spectra of *α*S fibrils aggregated in the presence of varying concentrations of NaCl.

To investigate the effect of ionic strength on the amyloid formation, we aggregate monomeric *α*S in 10 mM Tris buffer (pD = 7.4) with increasing concentrations of NaCl (0–300 mM). AFM images show that *α*S fibrils prepared in up to 25 mM NaCl (henceforth ‘low-salt aggregation conditions’) have a ribbon-like morphology, while those prepared in more than 25 mM NaCl (henceforth ‘high-salt aggregation conditions’) are twisted and rod-like (Fig. 1A,B), similar to those observed recently in ref. 7 (see Fig. S1 for AFM images at all investigated salt concentrations). The UV-CD spectra (Fig. 1C) show a typical negative peak at ~218 nm irrespective of the ionic strength, and a positive peak that is blue-shifted from ~200 to ~195 nm for high-salt as compared to low-salt fibrils, in line with previously published UV-CD spectra of *α*S fibrils prepared in various salt concentrations^47^,^48^,^49^. Our XRD results (Fig. 1D) also indicate that we have fibrils similar to those studied in refs 7 and 50. To investigate the changes in molecular structure caused by differences in the ionic strength we employ 1D- and 2D-IR spectroscopy.

### 1D- and 2D-IR spectra of *α*S fibrils as a function of ionic strength: significant changes around [NaCl] = 25 mM

**Figure 2:**
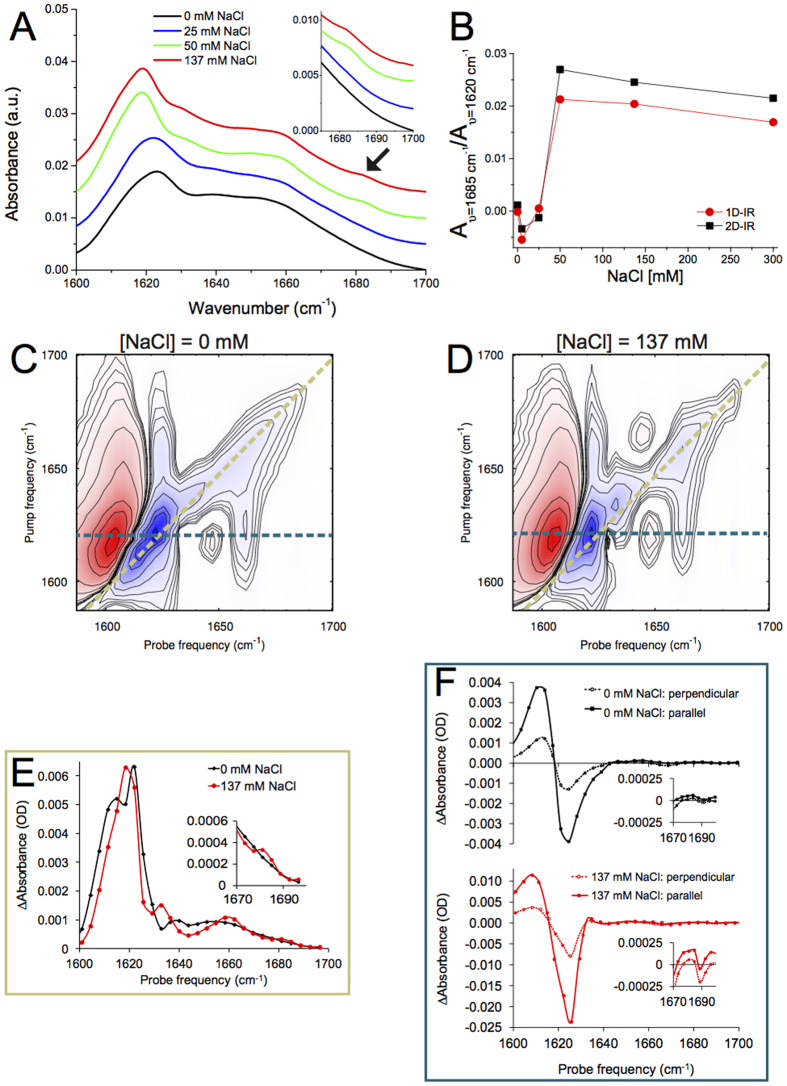
(**A**) 1D-IR (FTIR) spectra, (**B**) the 1685 cm^−1^ to 1620 cm^−1^ absorbance peak ratio, (**C**,**D**) 2D-IR spectra, (**E**) diagonal slices (along the yellow lines in (**C**) and (**D**)), and (**F**) slices at *ν*_p*ump*_ = 1620 cm^−1^ (along the blue lines in (**C**) and (**D**)) for *α*S fibrils aggregated with different concentrations of NaCl. Lines through the points in (**E**,**F**) are Catmull-Rom splines.

For all investigated fibrils, the IR spectra are dominated by a fibrillar *β*-sheet peak around 1620 cm^−1 ^31,51,52, and a peak at ~1657 cm^−1^, which is generally assigned to turns^51^,^53^ (see Fig. 2). However, the spectra of fibrils formed with [NaCl] > 25 mM are significantly different from those formed in [NaCl] < 25 mM, mainly by the appearance of a small peak at ~1685 cm^−1^ (see arrow in Fig. 2A, and the corresponding 2D-IR peak at (*ν*_probe_, *ν*_pump_) = (1685,1685) cm^−1^ peak in Fig. 2D), and by the disappearance of the ~1617 cm^−1^ shoulder superimposed on the broad 1623 cm^−1^ peak observed for low-salt fibrils. For high-salt fibrils this broad peak changes into two sharper peaks at ~1620 and ~1632 cm^−1^ (see also Fig. 2E). The spectral change is not gradual but discrete (as can be seen in Fig. 2B where the ratio between the absorption at 1685 and 1620 cm^−1^ is plotted), occurring at an onset NaCl concentration between 25 and 50 mM.

### Assignment of the IR spectra: evidence for a novel *β*-sheet normal mode

**Figure 3:**
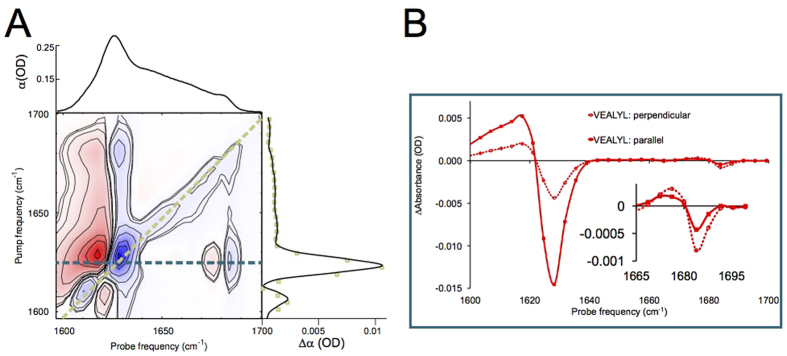
(**A**) 2D-IR spectrum, with (on top) the 1D-IR spectrum and (on the right side) the diagonal slice of the fibril formed by the hexapeptide VEALYL, that has an antiparallel orientation of *β*-strand monomers along the fibril axis, showing a canonical antiparallel *β*-sheet peak pattern with a perpendicular orientation of the low- with respect to the high-frequency modes, and (**B**) the slice at *ν*_p*ump*_ = 1620 cm^−1^. The cross peak at (*ν*_p*robe*_, *ν*_p*ump*_) = (1680, 1620) cm^−1^ with an anisotropy *R* = −0.21 ± 0.03 reveals that there is a 90 ± 11° angle between the modes that the low- and the high-frequency peaks are composed of.

The 1685 cm^−1^ peak is fundamentally different from the commonly observed^54^,^55^ high-frequency mode of antiparallel *β*-sheets. This becomes clear from the polarization dependence of the cross peak between the low- and high-frequency peak in the 2D-IR spectrum. The polarization dependence can be described by the anisotropy 
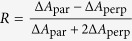
, with Δ*A*_par,perp_ the cross-peak intensity for parallel and perpendicular pump versus probe polarization, respectively. In a canonical antiparallel *β*-sheet the transition dipole moment (TDM) of the high-frequency mode is perpendicular to the TDM of the low-frequency (<1640 cm^−1^) *β*-sheet mode, which corresponds to 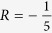,^32^ which we also measure for the hexapeptide VEALYL (*R* = −0.21 ± 0.03) that forms fibrils composed of antiparallel intermolecular *β*-sheets (Fig. 3), similar to what previous studies^56^,^57^,^58^ found for antiparallel *β*-sheets. Surprisingly, for high-salt *α*S fibrils we find R = −0.06 ± 0.09, indicating that the conventional antiparallel *β*-sheet assignment does not apply for *α*S, in line with the ss-NMR findings^7^,^10^,^18^,^19^,^20^,^21^,^22^,^23^ that *α*S are in-register (*i.e.* not composed of antiparallel intermolecular *β*-sheets).

The high-frequency mode of high-salt *α*S fibrils can neither be assigned to in-register turns nor to loop regions, as was the case for the ~1685 cm^−1^ peak observed for amyloid fibrils formed by hIAPP^43^. *α*S Fibrils have a similar number of residues in turns for low- and high-salt fibrils, according to the UV-CD spectra^59^ (Fig. 1C) and the NMR spectra measured on similar fibrils^7^,^10^, so it is unlikely that the appearance of the extra peak is due to a change in the amount of residues in loop regions. Also, proton-assisted insensitive nuclei (PAIN) cross-polarization spectra of *α*S fibrils show that the loop regions have few contacts with the loops in neighboring *α*S molecules^10^, so it is also unlikely that high-salt *α*S fibrils would contain the highly-ordered loop regions that are required to absorb at such high frequencies: if only because the PAIN spectra show that they are definitely not more ordered than the loop region in low-salt fibrils.

### Structural interpretation of the change in the IR spectra with the novel assignment of the high-frequency mode from spectral calculations: *α*S forms parallel, extended fibrils in low-salt buffers, and antiparallel, tightly packed fibrils in high-salt buffers

**Figure 4:**
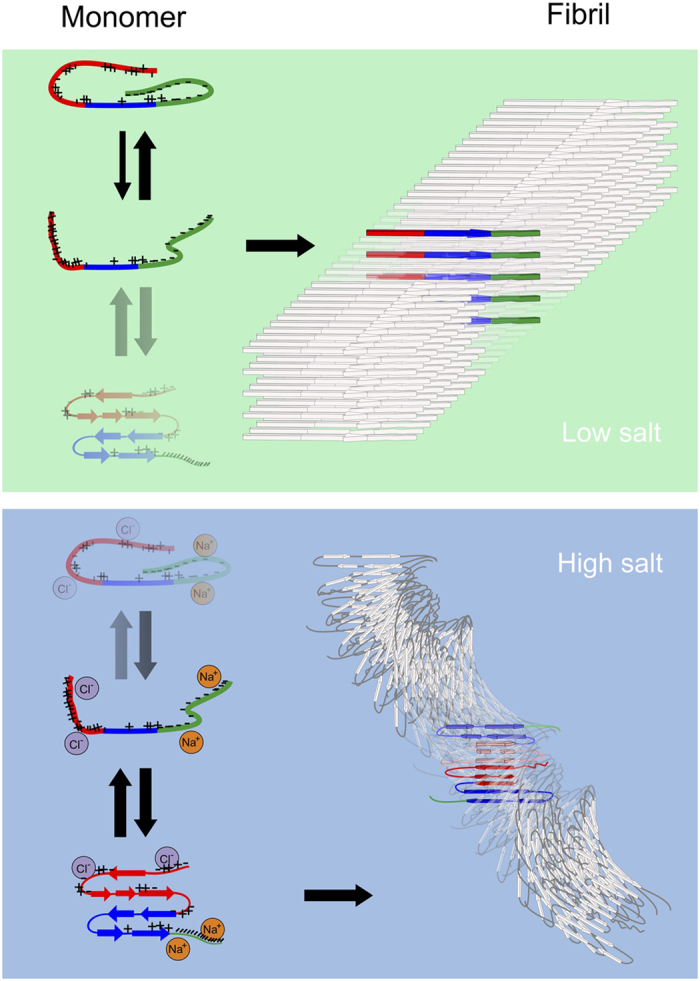
Proposed structures that explain the different IR spectra and AFM images for low- versus high-salt fibrils. By adding ions to the solution of *α*S monomers the conformational equilibrium is changed from a situation where most *α*S molecules are in a conformation in which the oppositely charged C- and N-terminus shield the hydrophobic NAC region, to a situation in which the charge interaction is screened due to the ions^6^,^13^,^14^,^16^,^17^. This leads to a more exposed NAC region, in which *α*S can transiently adopt intramolecular *β*-sheet structure^15^, with a potential high fibrillization propensity after a 90° rotation of the hydrogen bonds that has been observed before in aggregating proteins^69^. The width of the low-salt fibrils, as observed with AFM, is similar to the length of an extended *α*S monomer, and the low-frequency shoulder (~1617 cm^−1^) in the IR spectra is only observed for a parallel orientation of hydrogen-bonded *β*-sheets. We therefore hypothesize that in a low-salt buffer the C- and N-terminus shielded monomer conformation is in equilibrium with an extended conformation that has a stronger fibrillization propensity than the shielded conformation (albeit much less strong as compared to the intramolecular *β*-sheet conformation that is populated in a high-salt buffer, as judged from the much slower aggregation rate of low-salt fibrils). The extended monomer conformation leads to an extended and parallel fibril structure. This hypothesis also explains the fact that the low-salt fibrils exhibit no twist, whilst the high-salt fibrils are composed of two entwined twisting protofibrils (as also observed with cryo-electron microscopy^83^), as both charge^84^ and size^85^ are known to influence the twisting properties of protofibrils.

More probably, the spectral differences for varying salt concentrations reflect a difference in the intramolecular structure of *α*-synuclein within the fibril, from a more extended form for low-salt fibrils, to a more tightly-packed, probably antiparallel intramolecular conformation for the high-salt fibrils (see Fig. 4).

Spectral calculations show that a new high-frequency normal mode appears when hydrogen-bonded fibrillar intermolecular *β*-sheets are stacked in the direction perpendicular to the fibril axis. In the case of a single parallel sheet (see Fig. S2A for the structure, Fig. S3A for the calculated spectrum and Fig. S4A for the normal mode analysis), almost all intensity goes into low-frequency modes that have TDMs parallel to the hydrogen bonds between the backbone amide groups^54^; in the case of a single antiparallel sheet (see Figs S2B, S3A and S4B,C) almost all intensity goes into one low-frequency mode with a TDM in the direction of the backbone hydrogen-bonds, and into a high-frequency mode with a TDM that is perpendicular to this mode, in the direction of the *β*-strands^54^,^55^. However, when the in-register intermolecular *β*-sheets are stacked closely enough, a different high-frequency mode appears, that absorbs at approximately the same frequency as the high-frequency mode of *β*-sheets composed of *β*-strands that are oriented in an antiparallel fashion. This high-frequency mode is of a fundamentally different nature than the canonical high-frequency mode of an antiparallel *β*-sheet: the TDM of the high-frequency mode of stacked *β*-sheets is parallel to the fibril axis (rather than perpendicular to the fibril axis; see Figs S3B and S4D). In addition, besides this mode and the canonical low-frequency parallel *β*-sheet mode (Fig. S4E), new intense low-frequency modes appear with TDMs either parallel to the *β*-strands (Fig. S4F), or in the direction in which the intermolecular *β*-sheets are stacked (Fig. S4G).

The above-mentioned cross-peak anisotropy of −0.06 ± 0.09 in the spectrum of high-salt *α*S fibrils can be well explained by the presence of multiple orthogonal modes contributing to the low-frequency peak. If each of the two diagonal peaks giving rise to the cross peaks were due to a single normal mode, the cross-peak anisotropy would be −0.2 or 0.4 for perpendicular and parallel TDMs, respectively, while modes at intermediate TDM angles would give rise to intermediate *R* values. Because it is unlikely that modes at intermediate angles are present in the highly symmetric fibril structure (which we also see in the spectral calculations), we think that the observed cross-peak anisotropy reflects the fact that the contributing absorption peaks are composed of multiple modes with orthogonal TDMs as this also results in an intermediate *R* value.

### Spectral calculations on *in silico* constructed *α*S-like fibrils reproduce the experimentally observed normal modes

**Figure 5:**
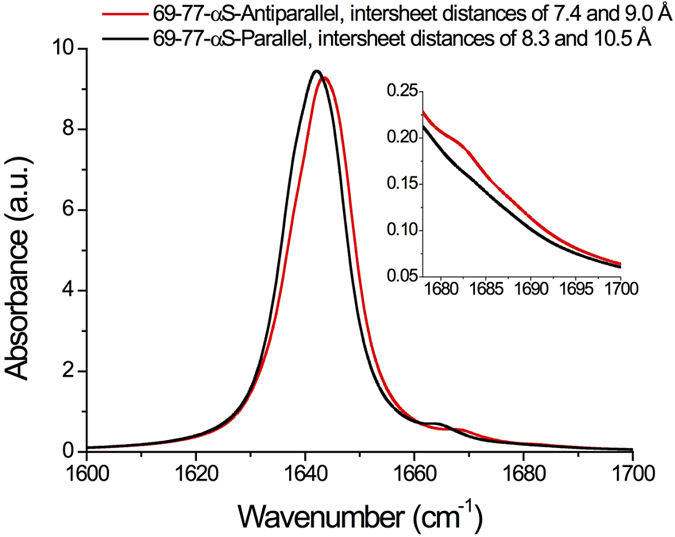
Calculated IR spectra for two *in silico* constructed *α*S-like fibril structures: (red) a fibril composed of 10 intermolecular *β*-sheets laterally stacked in an antiparallel fashion with an inter-sheet distance of 7.4 and 9.0 Å, and (black) a fibril composed of 10 intermolecular *β*-sheets laterally stacked in a parallel fashion with an inter-sheet distance of 8.3 and 10.5 Å.

Such a low-frequency peak composed of modes with multiple orthogonal TDMs is observed in spectral calculations on an *in silico* constructed *α*S-like fibril based on the unit cell of the fibril formed by the 69–77 segment of wild-type *α*S that contains intermolecular *β*-sheets that have an antiparallel orientation with respect to each other^22^ (see Fig. S2C,D). Assuming such a structure, the high-frequency mode visible in the spectra of high-salt fibrils can be well reproduced (see Fig. 5). In this calculation, the unit cell of the 69–77 segment fibril (PDB ID: 4RIK) has been extended into 10 stacked intermolecular *β*-sheets^60^, and 15 peptides long in the fibril direction (for more than ~15 peptides in this direction, the spectra converge, see Fig. S5). The main distances found in the XRD-spectra are present in the structure thus obtained (see Fig. S2E,F). The underestimation of the calculated frequency splitting with respect to the experimentally observed splitting has been noted before for the transition dipole coupling (TDC) approximation we employ here^61^,^62^,^63^, but for a qualitative understanding of IR spectra this poses no problems^51^,^54^,^64^,^65^,^66^.

If we slightly expand the structure in order to match the distances in the low-salt XRD spectrum (inter-sheet distances of 8.3 and 10.5 Å) and stack the hydrogen-bonded sheets in a parallel fashion, we obtain a calculated IR spectrum that, like the experimental low-salt IR spectrum, does not show the ~1685 cm^−1^ peak (see Fig. 5). The low-frequency region of the experimental low-salt fibril spectra (most clearly visible in the diagonal slices of the 2D-IR spectra in Fig. 2E) is best also reproduced for a parallel orientation of the intermolecular *β*-sheets: for such structures a weak low-frequency shoulder appears in the calculations, at ~5 cm^−1^ below the main fibrillar peak. If such parallel intermolecular *β*-sheets align as depicted in Fig. 4, the width of the fibril is close to that observed with AFM (see Fig. S1). We therefore hypothesize that the low-salt fibrils are composed of intermolecular *β*-sheets with a parallel orientation.

An investigation of the influence of the orientation, distance and number of laterally stacked intermolecular *β*-sheets on the calculated spectra of fibrils formed by three model hexapeptides (GGVVIA, NNQQNY and SNQNNF, see Supporting Information) shows that these effects are not unique for the *in silico* constructed *α*S-like fibrils, but that generally for in-register amyloid fibrils (I) a high-frequency peak appears when they are composed of hydrogen-bonded *β*-sheets that are closely packed, and (II) a low-frequency shoulder appears when they are composed of hydrogen-bonded *β*-sheets stacked in a parallel fashion (in the direction perpendicular to the fibril axis).

Interestingly, for fibrils composed of laterally stacked antiparallel intermolecular *β*-sheets (*i.e.* not in-register sheets) all the low-frequency modes remain parallel to the fibril axis, while the high-frequency mode remains in the direction of the *β*-strands; normal-mode orientations similar as for isolated antiparallel (intermolecular) *β*-sheets (Fig. S8). This explains why the fibril formed by VEALYL (composed of such laterally stacked antiparallel intermolecular *β*-sheets) has a similar 2D-IR spectrum and cross peak anisotropy (Fig. 3) as an isolated antiparallel *β*-sheet^56^,^57^,^58^ (Figs S2B, S3A and S4B,C).

Because we observed a strong spectral influence of the number of stacked intermolecular *β*-sheets in the spectral calculations of the in-register fibrils (in line with previous studies^51^), we varied this parameter for the *α*S-like constructs described before, and found that the 4 peaks observed in the experimental high-salt fibrils could be best reproduced by stacking only 5 intermolecular *β*-sheets instead of 10 (see Fig. S8), suggesting that the most-pronounced peaks in the IR spectrum of fibrils may all originate from the *β*-sheets present in the *α*S fibrils (random-coil and turn peaks being too broad to observe), and that the two protofibrils that twist around each other (each composed of 5 intermolecular *β*-sheets) to form the 7 nm-thick fibril (Fig. 1) are vibrationally uncoupled, either because of the comparatively large distance separating them, or because they twist around each other without a well-defined phase relation.

## Discussion

From IR measurements (see Fig. S9) we find that the fibril structure does not change if we exchange the buffers of the fibrils with a different salt concentration after the fibrillization process is finished, in agreement with previous studies^7^. This implies that the effect of salt on the fibril structure must involve an influence on the monomeric (or oligomeric) conformational distribution during the nucleation and/or growth phase. The fact that we find evidence for more tightly packed intramolecular antiparallel *β*-sheet regions in fibrils grown under high-salt conditions may be related to the influence of charge on the conformation of *α*S monomers. In a low ionic strength solution *α*S is mainly in a conformation in which the hydrophobic non-A*β* component (NAC) region is shielded by the negatively charged C-terminus region that folds onto positively charged parts of the protein^16^,^17^. This presumably counteracts spontaneous aggregation^67^. Upon the addition of polycations *α*S unfolds and the NAC region is exposed^16^,^68^ (see Fig. 4). An increased exposure of the NAC region due to changes in pH 14,17, addition of polycations^14^,^16^, or changes in ionic strength (see Fig. 4) can increase the aggregation propensity of *α*S. The fact that the NAC region is predominantly shielded in low-salt conditions will probably result in amyloid aggregation via a different pathway than the aggregation pathway of monomers with an exposed NAC region: the high-salt conformer might promote an intramolecular hydrophobic collapse and subsequently the formation of antiparallel *β*-sheets, similar to the *β*-sheet formation observed in monomeric *α*S upon C-terminus binding of polyamines^15^. These antiparallel *β*-sheet conformers are likely to have a higher fibrillation propensity, possibly leading to full-grown fibrils consisting of intramolecular antiparallel *β*-sheets after a 90° rotation of the hydrogen bonds in the direction of the fibril axis, similar to the conformational transition that has been simulated and experimentally observed in the self-assembly of other proteins^69^.

## Conclusion

Our finding that even small variations in ionic strength (25 instead of 50 mM NaCl) can result in very different fibril structures could have significant physiological implications. The strong salt dependency described here is another example of the high sensitivity of *α*S fibrillization to aggregation conditions, besides previously described factors such as pH^70^, temperature^6^, and the presence and type of surface^71^ or phospholipid membrane^72^. The high sensitivity of *α*S to the aggregation conditions might contribute to the relatively frequent occurrence of contradicting findings in this field^73^. The mechanism proposed here should also be relevant for other amyloid forming proteins, since changes in ionic strength are known to affect the kinetics^74^,^75^ and structure^76^,^77^ of other amyloid forming proteins as well.

## Methods

### Terminology regarding the morphology

We use the following nomenclature to describe the different parts of the fibril structures: *β-strands* are covalently connected amino acids, that, by hydrogen bonding between backbone atoms of neighboring *β*-strands, can form hydrogen-bonded (fibrillar) *intermolecular β-sheets*. A *protofibril* is formed when multiple intermolecular *β*-sheets are connected via the backbones of the monomers that they are composed of, or (like in the right top panel of Fig. 4) when only a single intermolecular *β*-sheet is formed by the aggregated monomers. A *fibril* is formed when multiple protofibrils interact, e.g. by coiling around each other (like in the lower right panel of Fig. 4), to form a higher-order structure.

### Computational methods

The calculations have been performed using the Transition Dipole Coupling (TDC) model. This model is based on Coulomb-like coupling between the transition dipole moments of the local modes of the amide groups, according to


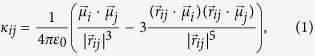


with *ε*_0_ the dielectric constant, 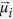 the transition-dipole of peptide bond *i*, and 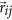 the vector connecting dipole *i* and *j*. The model has extensively been described before^64^,^65^,^78^,^79^.

From the calculated normal modes we calculate the IR spectrum as follows (assuming homogeneous line broadening for the individual normal modes):


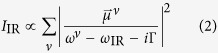


with 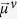 the transition dipole moment of mode *v, ω*^*v*^ the eigenvalues of the Hamiltonian (that give the normal-mode frequencies), *ω*_IR_ the frequency of the IR field, and Γ the linewidth. For all calculated spectra (unless noted differently) Γ = 5 cm^−1^ and the gas phase amide-I frequency is set to 1660 cm^−1^.

### Sample preparation

#### Expression, purification and labeling of *α*S variants

The *α*S used in this study was expressed in *Escherichia coli* strain BL21(DE3) using the pT7-7 expression plasmid as previously reported^80^.

#### Preparation of *α*S fibrils

Prior to aggregation, samples of *α*S in 10 mM Tris buffered at pH 7.4 were freeze-dried overnight in a ScanVac Coolsafe (Labogene) to remove H_2_O and subsequently, phosphate buffered saline (PBS; 137 mM NaCl, 3 mM KCl, 10 mM phosphate) or appropriate amounts of NaCl prepared in D_2_O was added to the dried protein in separate tubes to vary ionic strength in 10 mM Tris buffer. Thereafter, all protein samples were purified using a Nanosep^®^ 100 kDa molecular weight cut-off filter (Pall Corporation) to remove any pre-existing aggregates. The concentration of the resulting monomeric *α*S was measured in a NanoDrop 2000 UV-Vis spectrophotometer (Thermo Scientific) at 276 nm by using a molar extinction coefficient of 5600 cm^−1^M^−1^. The resulting purified samples did not show any absorbance beyond 320 nm (typically indicative of scattering) confirming the absence of any residual higher ordered aggregates. Finally, 150 *μ*M of purified *α*S monomers were shaken continuously in a Eppendorf Thermomixer^®^ at 1000 rpm, 37 °C until 90% conversion of monomers. For measurement of the percentage of monomer conversion, the aggregated suspensions were periodically aliquoted every 24 hours, ultracentrifuged at 10000 g to spin down aggregates, and the residual monomer concentration was estimated.

### Atomic Force Microscopy (AFM)

For the AFM measurements, 20 *μ*l of 10 *μ*M fibril suspensions obtained after aggregation were incubated on freshly cleaved mica (15 × 15 mm) for 5 minutes. Samples were then washed with pure D_2_O to remove salts and dried using a slow stream of N_2_ gas. Thereafter samples were kept in 37 °C for 1 hour to remove any residual D_2_O. AFM images were acquired in tapping mode on a Dimension 3100 Scanning Probe Microscope (Bruker) using NSG01 gold probes with a resonant frequency between 87–230 kHz and a tip radius <10 nm. Fibril heights and widths were determined using NanoScope Analysis v1.5 software.

### UV-Circular dichroism (UV-CD) spectroscopy

A Chirascan CD spectrometer was used to obtain UV-CD spectra of fibrils at a protein concentration of 10 *μ*M. Fibril samples were first purified using a 100 kDa cut-off filter to remove monomeric protein that can potentially affect the spectra. The UV-CD spectra were recorded between 200 to 250 nm with a step size of 1 nm and a scanning speed of 10 nm/min, using a 1-mm path-length cuvette at room temperature.

### X-ray diffraction (XRD)

A Philips X’Pert-MPD system with a Cu K*α* wavelength of 1.5418 Å in reflection *θ-θ* mode was used to analyze the structure of the fibrils. The samples (prepared in appropriate buffers) were deposited on a monocrystal substrate cut at an angle non-parallel to surface, with a beam stop mounted on top of the sample. During the measurements the sample was rotated at a speed of 4 s/revolution. The diffractometer was operated at 40 kV and 40 mA at a 2*θ* range of 2–40°, employing a step size of 0.025°.

### IR spectroscopy

The IR cells were prepared by pipetting 7.5 *μ*L of fibril solution in between two CaF_2_ windows that are separated by a 50 *μ*m teflon spacer. The 1D-IR spectra have been measured on a Bruker Vertex 70, with 32 scans and a resolution of 1 cm^−1^. The 2D-IR spectra have been measured on a setup described in detail before^81^. Briefly, at a repetition rate of 1 KHz, 3 mJ pulses with a central wavelength of 794 nm are converted in an optical parametric amplifier into mid-IR (~20 *μ*J, ~6100 nm) pulses with a spectral full width at half max (FWHM) of 150 cm^−1^. This beam is split into a probe and reference beam (each 5%), and a pump beam (90%) that is aligned through a Fabry-Pérot interferometer by which it is narrowed to a FWHM of 10 cm^−1^. The pump and probe beam are overlapped in the sample with a 1.5 ps delay in a ~200 *μ*m focus, and the Δabsorption (Δ*α*) is recorded after dispersion by a OrielMS260i spectrograph onto a 32 pixel MCT array with a resolution of 3.9 cm^−1^. The background is subtracted by comparing the Δ*α* at (*t*_probe_ − *t*_pump_) = 1.5 and −10 ps. All IR spectra were measured at room temperature, and all presented 2D-IR spectra are (unless noted differently) measured with a perpendicular orientation of pump versus probe beam (obtained by putting the probe beam at a 45° degree angle with the pump beam, and selecting the probe beam component that is either perpendicular or parallel to the pump beam using a polarizer after the sample, in order to avoid beam pointing differences for the two polarizations). The anisotropy 
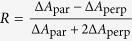
 of the cross peaks is correlated with the relative orientation of the transition dipole moments *θ* of the peaks with *R* = (3cos(*θ*)^2^ − 1)/5. The 2D-IR spectra were typically recorded by measuring 10 scans of each approximately 1 hour; the standard deviations *σ*_R_ and *σ*_*θ*_ were calculated by determining R and *θ* for each scan individually. In order to minimize scattering contributions the average of 2 PEM-induced pump delays was measured such that the interference between the scattered pump beam and the probe beam has a 180° phase in one delay with respect to the other delay (analogous to the scatter reduction presented in ref. 82 where a wobbler is used for the same purpose).

## Additional Information

**How to cite this article**: Roeters, S. J. *et al*. Evidence for Intramolecular Antiparallel Beta-Sheet Structure in Alpha-Synuclein Fibrils from a Combination of Two-Dimensional Infrared Spectroscopy and Atomic Force Microscopy. *Sci. Rep.* **7**, 41051; doi: 10.1038/srep41051 (2017).

**Publisher's note:** Springer Nature remains neutral with regard to jurisdictional claims in published maps and institutional affiliations.

## Supplementary Material

Supporting Information
