# Supplementary material for: Evidence for Intramolecular Antiparallel Beta-Sheet Structure in Alpha-Synuclein Fibrils from a Combination of Two-Dimensional Infrared Spectroscopy and Atomic Force Microscopy
Source: Sci Rep. 2017 Jan 23;7:41051. doi: 10.1038/srep41051 (PMC5253669; doi:10.1038/srep41051)
Supplement: Supporting Information [file srep41051-s1.pdf]

Supporting information for: Evidence for Intramolecular Antiparallel Beta-Sheet Structure in Alpha-Synuclein Fibrils from a Combination of Two-Dimensional Infrared Spectroscopy and Atomic Force Microscopy

Steven J. Roeters[1,*,+], Aditya Iyer[2,+], Galja Pletikapić[2], Vladimir Kogan[3], Vinod Subramaniam[2,4], and Sander Woutersen[1,]**

[1]Van 't Hoff Institute for Molecular Sciences, University of Amsterdam, Science Park 904, 1098 XH Amsterdam, The Netherlands
[*]s.j.roeters@uva.nl
[2]Nanoscale Biophysics Group, FOM Institute AMOLF, Science Park 104, 1098 XG Amsterdam, The Netherlands
[3]Dannalab BV, Wethouder Beversstraat 185, 7543 BK Enschede, The Netherlands
[4]Vrije Universiteit Amsterdam, De Boelelaan 1105, 1081 HV Amsterdam, The Netherlands
[**]s.woutersen@uva.nl
[+]These authors contributed equally to this work.

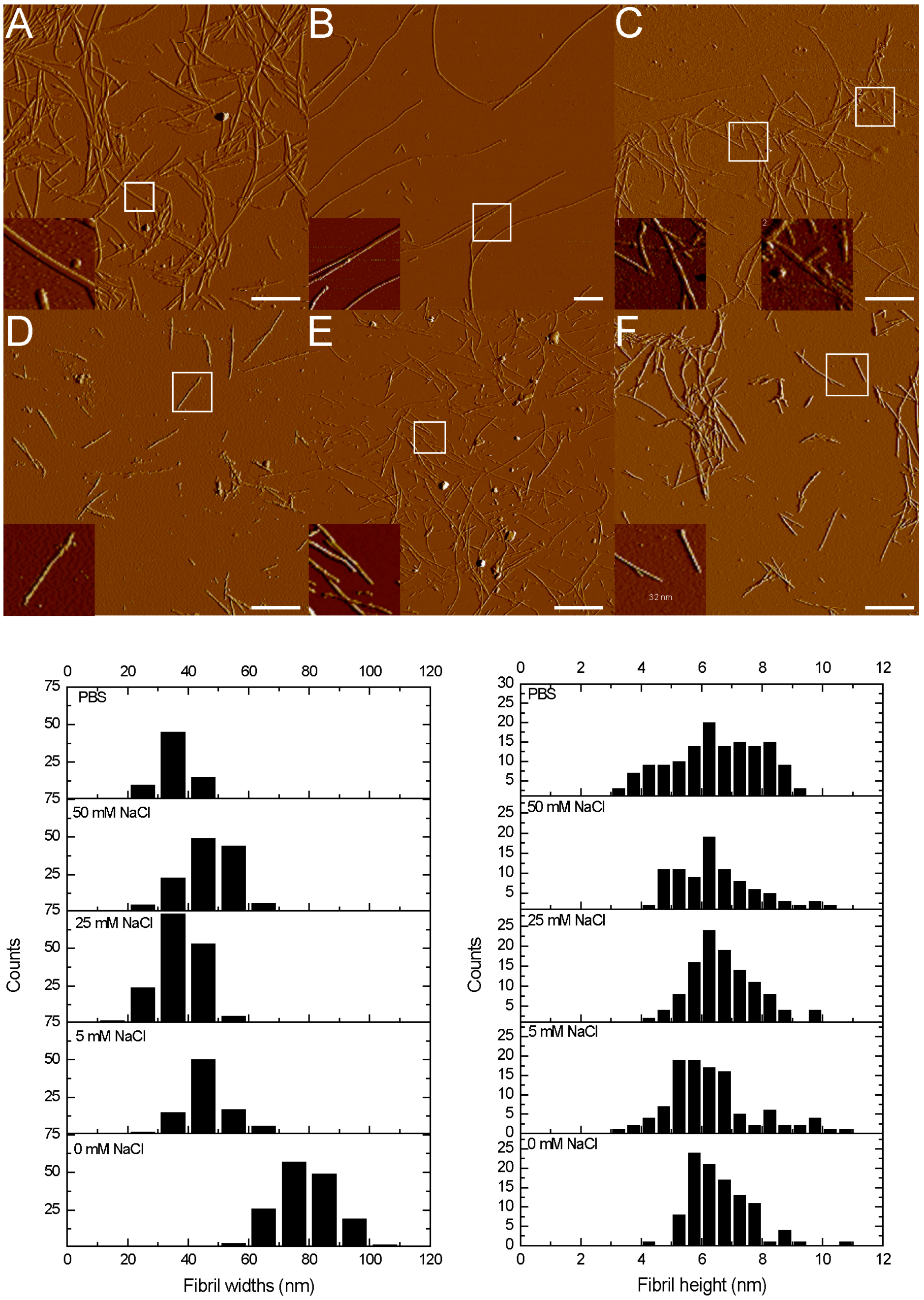

[NaCl]	N total	Mean fibril width (nm)	Standard Deviation	Median
0 mM NaCl	158	74	11	70
5 mM NaCl	90	42	8	40
25 mM NaCl	157	32	8	30
50 mM NaCl	128	42	10	40
137 mM NaCl*	70	31	6	30

[NaCl]	N total	Mean fibril height (nm)	Standard Deviation	Median
0 mM NaCl	102	6.67	1.12	6.43
5 mM NaCl	108	6.38	1.54	6.07
25 mM NaCl	115	6.73	1.17	6.56
50 mM NaCl	96	6.60	1.68	6.32
137 mM NaCl*	135	6.61	1.80	6.72

Figure 1. Representative AFM images at different ionic strengths: The top panels are amplitude images obtained upon aggregation of WT-αS in 10 mM Tris buffer with 0 mM NaCl (A), 5 mM NaCl (B), 25 mM NaCl (C), 50 mM NaCl (D), 100 mM NaCl (E) and PBS buffer* (137 mM NaCl, 3mM KCl and 10 mM phosphate) (F), at 37 °C with constant shaking at 1000 rpm. Insets are contrast enhanced for clarity. Scale bars are 10 μm. Panels B and C contain both wide and thin fibrils while panels D, E and F contain only thin fibrils ($\sim$30-40 nm wide). Fibrils heights and widths were obtained from AFM height images. Note that the reported fibril widths are actually convolutions of the actual fibril widths and the AFM tip width. As this effect is not present in the height measurement, and we assume the high-salt fibrils are as high as they are wide, we estimate that approximately 30 nm of the reported widths is due to the AFM tip width. Hence, the actual width of no-salt fibrils would be $\sim$50 nm, equal to the length of an extended αS molecule. Therefore the AFM data is consistent with a parallel and extended orientation of αS molecules in low-salt fibrils.

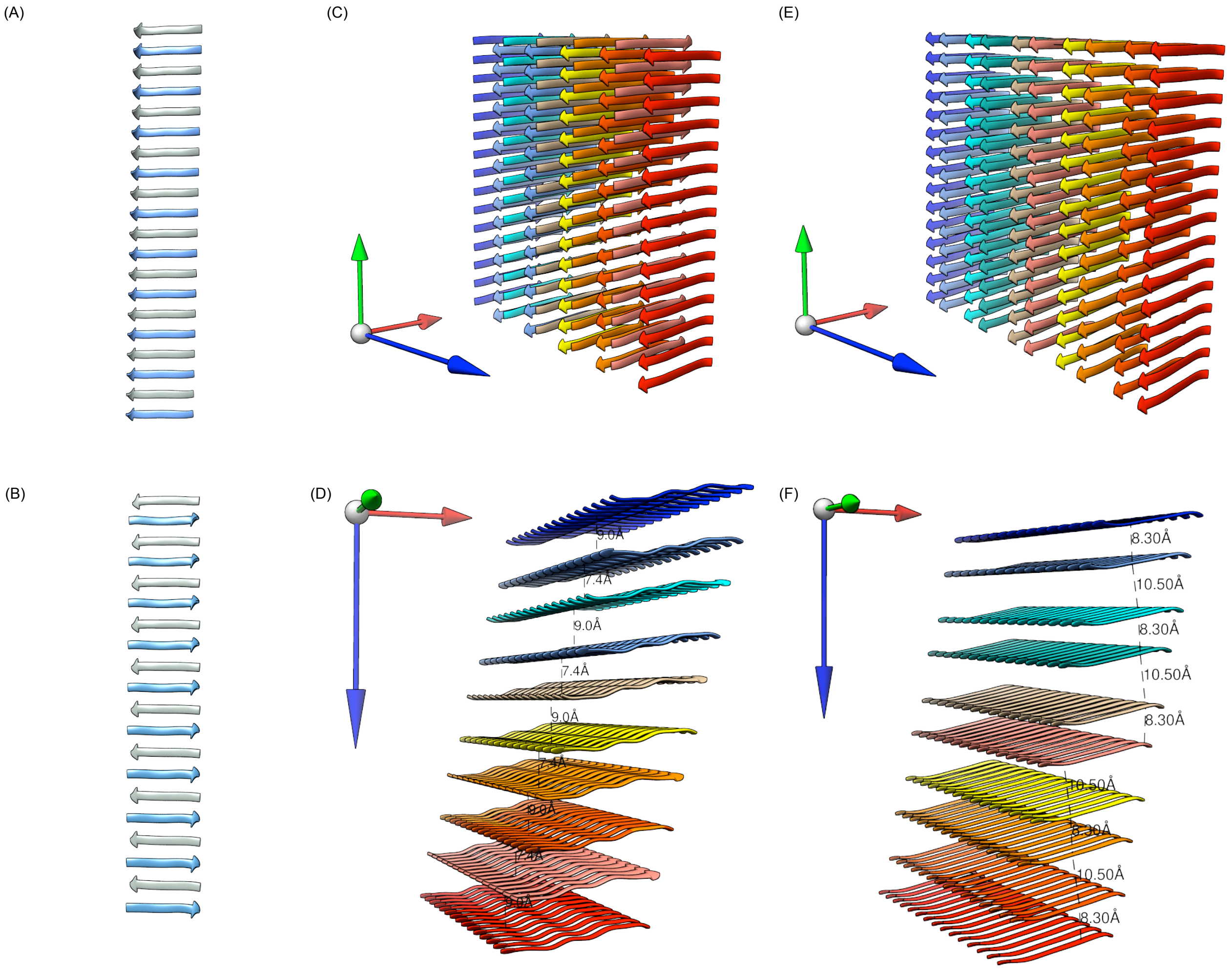

Figure 2. (A) One fibrillar parallel intermolecular β-sheet composed of SNQNNF peptides, (B) one fibrillar antiparallel intermolecular β-sheet, composed of VEALYL peptides, (C,D) αS-like structures used in the spectral calculations, created by stacking 10 hydrogen-bonded in-register fibrillar sheets in an antiparallel fashion with an inter-sheet spacing of 7.4 and 9.0 Å — as obtained by expanding the unit cell of the fibrils formed by the 69-77 segment of αS (PDB entry: 4RIK), and (E,F) an expanded and parallel version of this structure with inter-sheet spacing of 8.3 and 10.5 Å. In each case, the green arrow indicates the fibril axis

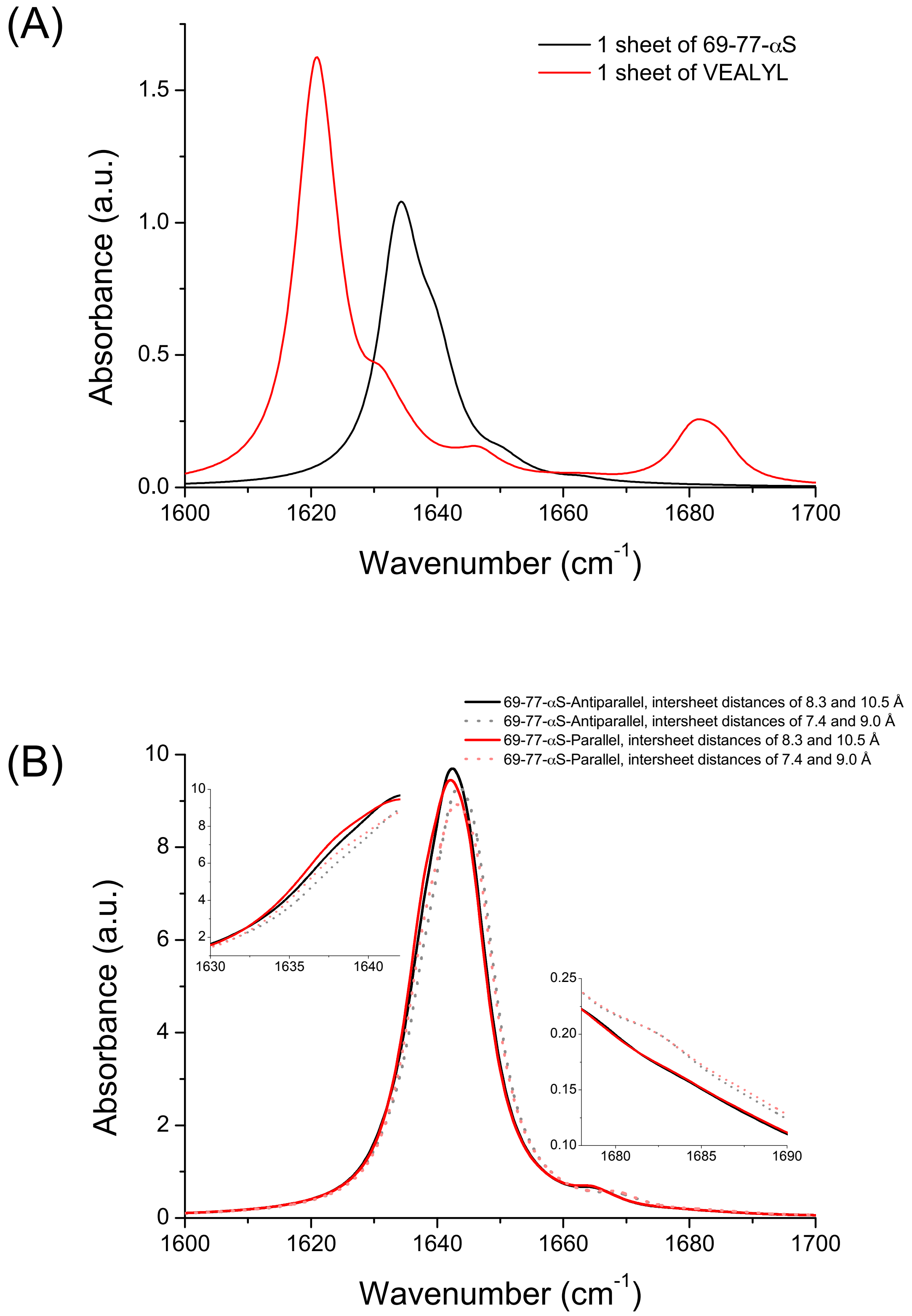

Figure 3. Calculated spectra (A) for single intermolecular β-sheets composed of antiparallel β-strands (VEALYL) and parallel β-strands (69-77αS), and (B) for structures constructed into 10 stacked intermolecular β-sheets, based on the unit cell of PDB entry 4RIK, the fibril of the 69-77 segment of αS, for 4 possible αS conformations.

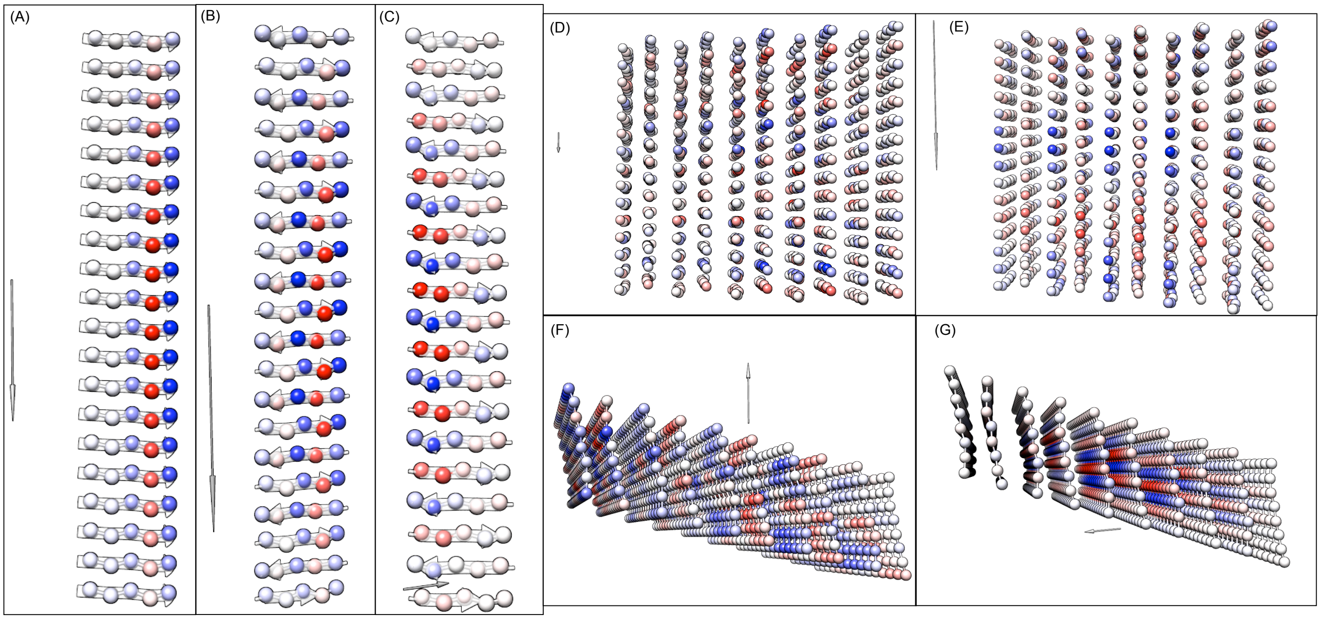

Figure 4. Transition-dipole moments (white arrows) and local-mode phases (red and blue spheres) of (A) the 'parallel β-sheet mode' of a single parallel intermolecular β-sheet of SNQNNF formed by 20 β-strands, with a frequency of 1630.08 cm^{-1} (as the theory dictates[1,2], there is no high-frequency mode with a significant intensity for such a parallel β-sheet), (B) the eigenmode with a frequency of 1620.83 cm^{-1} (with mainly in-phase oscillations with neighboring amide groups to which they are hydrogen bonded, leading to a transition dipole moment (TDM) of the eigenmode that is parallel to the hydrogen bonds that form the β-sheet, therefore known in the literature[3] as the 'parallel β-sheet mode') of a single antiparallel intermolecular β-sheet of VEALYL formed by 20 β-strands, (B) the eigenmode with a frequency of 1685.91 cm^{-1} (known in the literature[3] as the 'perpendicular β-sheet mode', which can be seen from the in-phase oscillations along the β-strands; perpendicular to the direction of the hydrogen bonds that form the β-sheet) of a single antiparallel intermolecular β-sheet of VEALYL formed by 20 β-strands, (D) the eigenmode with a frequency of 1682.5 cm^{-1} (dominated by in-phase oscillations of amide groups with their neighbors in neighboring hydrogen-bonded β-sheets, leading to a TDM parallel to the fibril axis), (E) the eigenmode with a frequency of 1645.42 cm^{-1} (the strongest mode in the calculated IR spectrum, dominated by in-phase oscillations of amide groups along the fibril axis and with their neighbors in neighboring hydrogen-bonded β-sheets, leading to a TDM along the fibril axis), (F) the eigenmode with a frequency of 1642.36 cm^{-1} (with in-phase oscillation along the fibril axis and with neighboring amide groups in the same β-strand, leading to a TDM in the direction of the β-strands), and (G) the eigenmode with a frequency of 1630.81 cm^{-1} (dominated by in-phase oscillations of amide groups with their neighbors in neighboring stacked hydrogen-bonded β-sheets and with neighbors in the fibril direction, leading to a TDM in the stacking direction) of the *in silico* constructed αS-like antiparallel structure that matches with the distances found in the high-salt XRD spectra, as described in the main text.

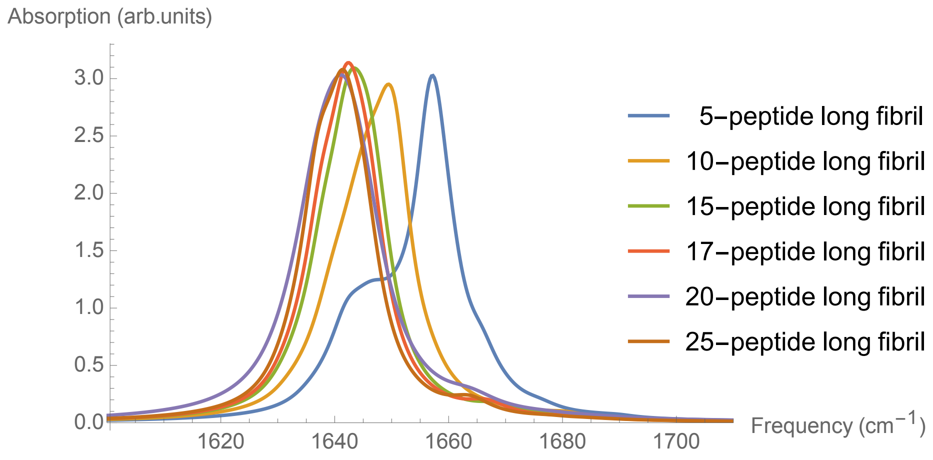

Figure 5. Calculated spectra for different numbers of β-strands in the fibril direction, for fibrils composed of 10 stacked intermolecular β-sheets.

Generalization of the spectral assignment based on calculations: are the modes present in the αS-like structures generally found in in-register fibrillar structures?

To study whether it is a general phenomenon that for amyloid fibrils (I) a high frequency peak appears when they are composed of hydrogen-bonded β-sheets that are closely packed, and that (II) a low-frequency shoulder appears when they are composed of hydrogen-bonded β-sheets stacked in a parallel fashion (in the direction perpendicular to the fibril direction), we investigate the influence of the orientation, distance and number of stacked hydrogen-bonded β-sheets on the calculated spectra of fibrils formed by three model hexapeptides: GGVVIA, NNQQNY and SNQNNF (see Figure S6B-D). The low-frequency shoulder ($\sim$1617 cm^{-1}) is indeed only present in fibrils with a parallel intermolecular β-sheet orientation, whilst the high-frequency peak ($\sim$1683 cm^{-1}) seems to be determined mainly by the number of stacked hydrogen-bonded β-sheets and their distance (see Figure S7), whilst being insensitive to whether the hydrogen-bonded β-sheets are parallel or antiparallel with respect to each other (the hydrogen-bonded β-sheets are stacked in a parallel fashion in SNQNNF, while they have an antiparallel orientation in GGVVIA and NNQQNY). This result is similar to what we find for the *in silico* constructed αS-like structures (see Figure S3B). The dependency on the number of stacked β-sheets and the intersheet-distance of the high-frequency peak can be understood by inspection of the phase of the associated $\sim$1685 cm^{-1} mode of e.g. GGVVIA (see Figure S6E): the amide-groups oscillate in phase with their neighbors in the neighboring hydrogen-bonded β-sheets. This is in contrast to most other normal modes with significant intensity, in which amide groups oscillate mainly in phase with their neighbors in the *same* hydrogen-bonded β-sheet (see e.g. Figure S6F). For all hexamers studied here, the modes around 1620 cm^{-1} increase in frequency with decreasing inter-β-sheet distance and with an increasing number of intermolecular β-sheets, in line with previous work[4].

References

1. Miyazawa, T. Perturbation Treatment of the Characteristic Vibrations of Polypeptide Chains in Various Configurations. *J. Chem. Phys.* **32**, 1647 (1960).
2. Barth, A. & Zscherp, C. What vibrations tell about proteins. *Q. Rev. Biophys.* **35**, 369–430 (2002).
3. Ganim, Z. *et al.* Amide I two-dimensional infrared spectroscopy of proteins. *Acc. Chem. Res.* **41**, 432–441 (2008).
4. Karjalainen, E.-L., Ravi, H. K. & Barth, A. Simulation of the Amide I Absorption of Stacked β-Sheets. *J. Phys. Chem. B.* **115**, 749–757 (2011).
5. Slotta, U. *et al.* Spider Silk and Amyloid Fibrils: A Structural Comparison. *Macromol. Biosci.* **7**, 183–188 (2007).
6. Sawaya, M. R. *et al.* Atomic structures of amyloid cross-β spines reveal varied steric zippers. *Nature* **447**, 453–457 (2007).

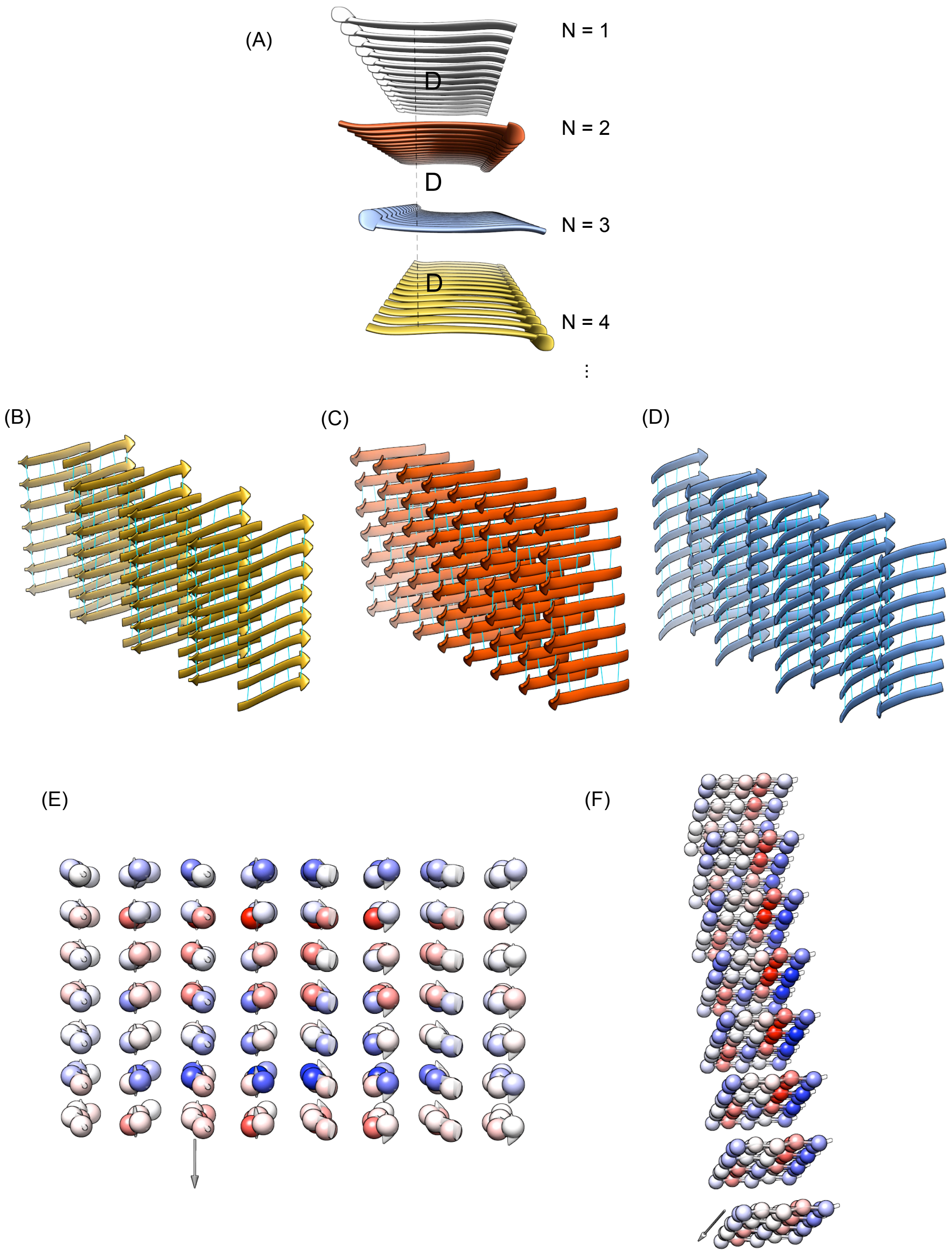

Figure 6. (A) General structure used to investigate whether the results of the spectral calculations are generic for all amyloid fibril structures, with N hydrogen-bonded (depicted as blue lines) intermolecular β-sheets at inter-sheet distances D. The influence of N and D on the spectrum was systematically studied for the fibrils formed by the hexapeptides (B) NNQQNY (PDB entry: 1YJO) , (C) SNQNNF (PDB entry: 2OL9) and (D) GGVVIA (PDB entry: 2ONV), here all depicted with 7 peptides in the fibril direction and 8 stacked hydrogen-bonded β-sheets. (E) The high-frequency ($\sim$1685 cm^{-1}) fibrillar β-sheet mode of the fibril formed by the GGVVIA. Note that the amide groups oscillate in phase with their neighbors in the neighboring intermolecular β-sheets, in contrast to most other modes with considerable intensity to which the local modes contribute by oscillating in phase with their neighbors in the same hydrogen-bonded β-sheet, like for (F) the most-intense low-frequency mode ($\sim$1640 cm^{-1}) of SNQNNF, that like most low-frequency fibrillar modes has in-phase oscillation in the fibril direction, sometimes in combination with in-phase oscillations also in the lateral direction; with neighboring amide groups in the neighboring hydrogen-bonded β-sheets.

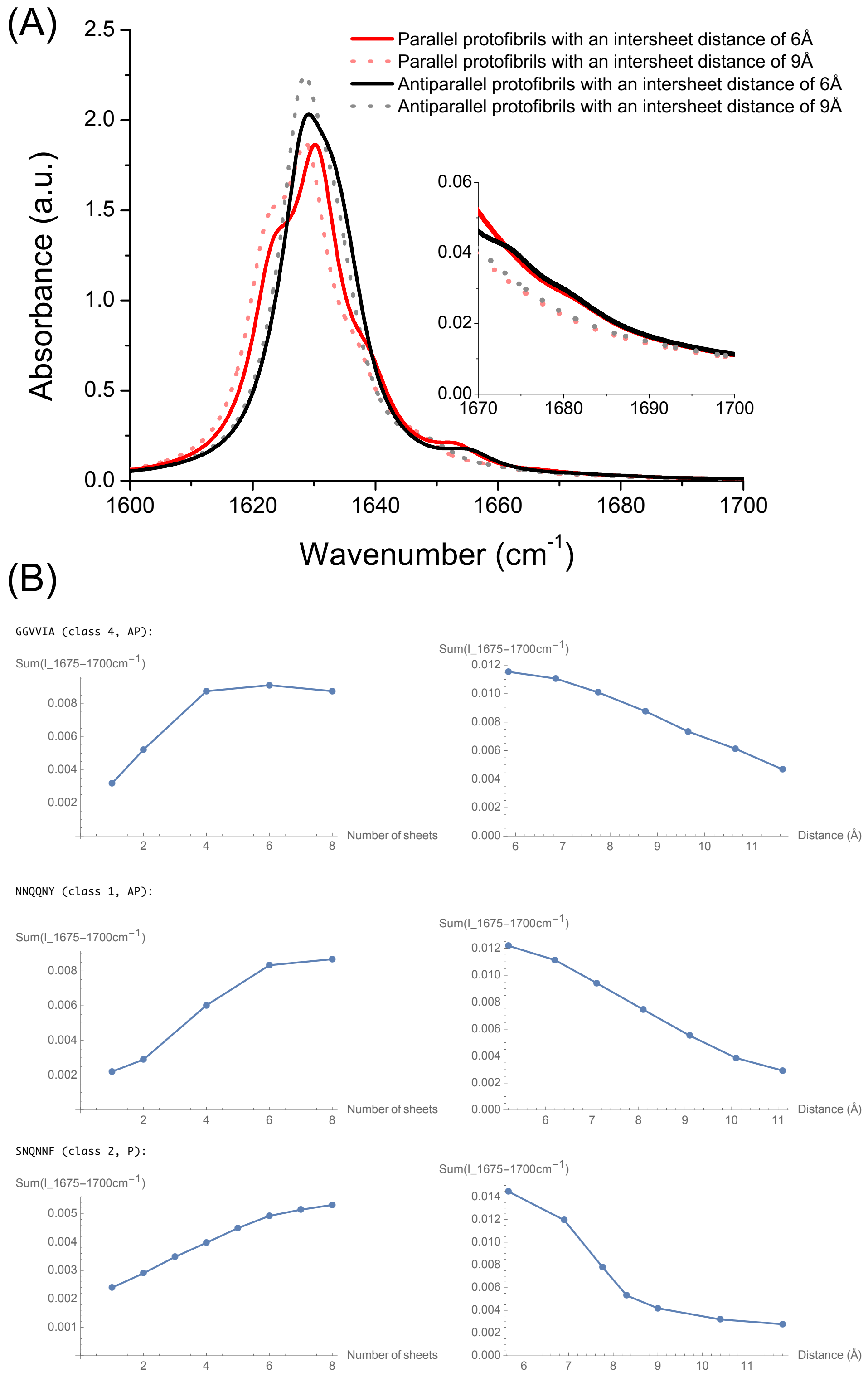

Figure 7. (A) Calculated spectra for 4 stacked sheets of 14 peptides with varying inter-sheet distances, of SNQNNF (stacked in a parallel fashion) and NNQQNY (stacked in a antiparallel fashion), (B) Ratio of the intensity in the 1675-1700 cm^{-1} region to the total (1600-1700 cm^{-1}) amide-I intensity as a function of the number of intermolecular β-sheets and their spacing distance. The lowest inter-sheet distances presented here are known to occur in nature, *e.g.* in the so-called nanofibrils in the silk gland of the golden silk orb-weaver spider (5.3Å) [5].

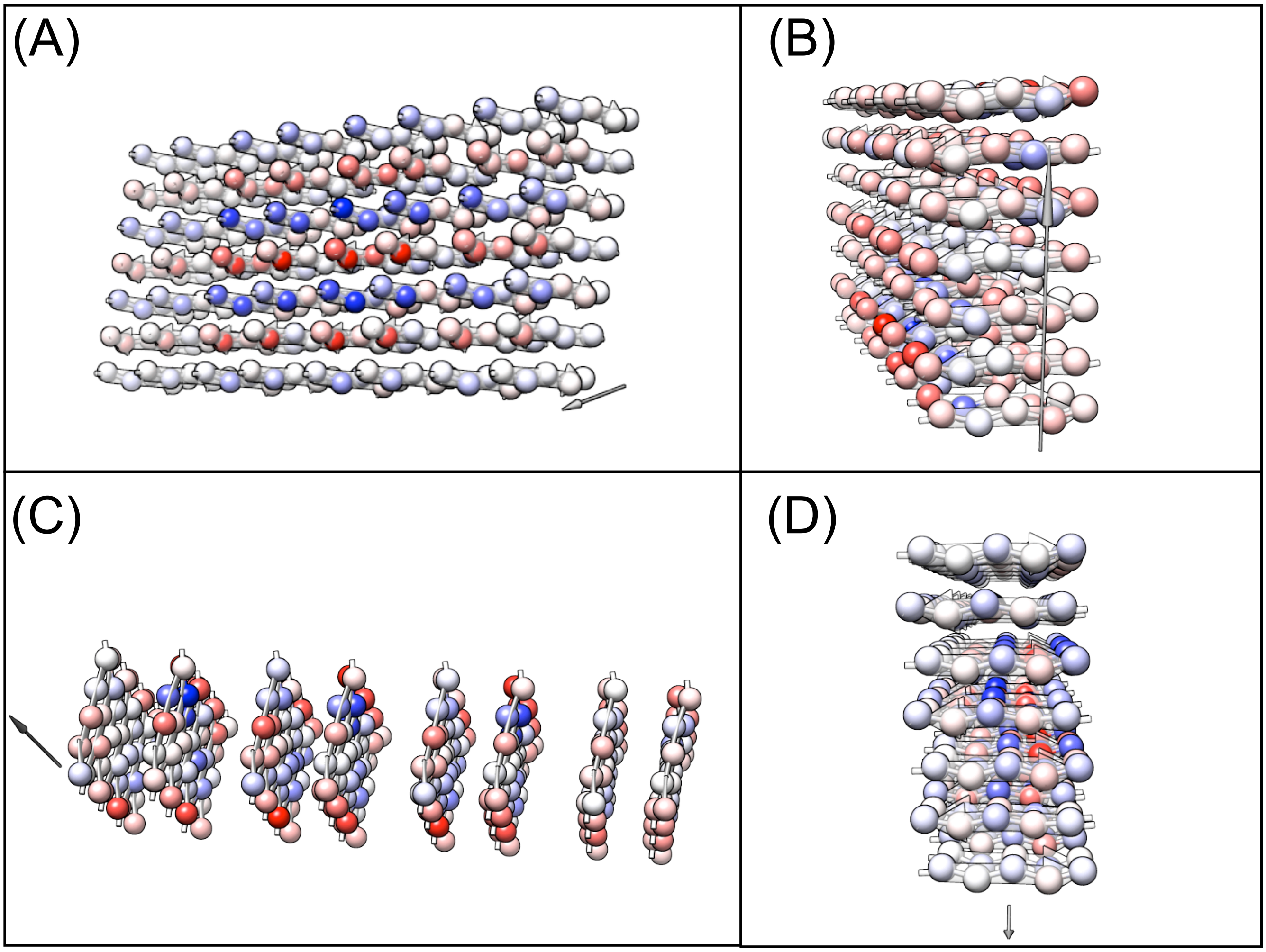

Figure 8. Calculated normal modes for VEALYL, with the transition dipole moments (TDMs) depicted by the grey arrows, and the local mode contribution phases in red-blue, for: (A) the 1685.99 cm^{-1} normal mode in which the β-strands oscillate in phase, (B) the 1635.59 cm^{-1} normal mode caused mainly by inter-protofibril oscillations, (C) the 1634.32 cm^{-1} normal mode originating both from inter-protofibril and intermolecular β-sheet oscillations, and (D) the 1622.07 cm^{-1} normal mode, also caused mainly by inter-protofibril interactions. Interesting, as opposed to in-register fibrils, all low-frequency modes of significant magnitude have a TDM perpendicular to the fibril axis and all high-frequency modes have TDMs along the β strands. The calculation results presented here are performed on VEALYL structures modified to have a similar packing as the αS-like structures presented in Figure 4, in order to have the fairest comparison between antiparallel and parallel (*i.e.* in-register) intermolecular β-sheets; the X-ray crystal structure of VEALYL (PDB entry 2OMQ[6]) actually has every odd protofibril displaced by 10 Å in the β-strand direction, which does not significantly change the calculated normal modes — still all low-frequency modes of significant intensity lie along the fibril axis, while the high-frequency modes lie along the β-strands.

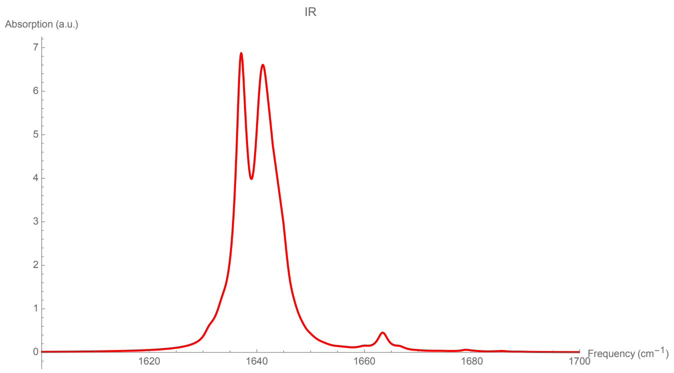

Figure 9. Calculated spectrum for structure constructed into 5 stacked hydrogen-bonded fibrillar β-sheets, based on the unit cell of PDB entry 4RIK, the fibril of the 69-77 segment of αS. The linewidth is set to 1 cm^{-1} to clearly show the four peaks. A similar peak sharpening can be expected for the highly crystalline structure that a fibril can adopt.

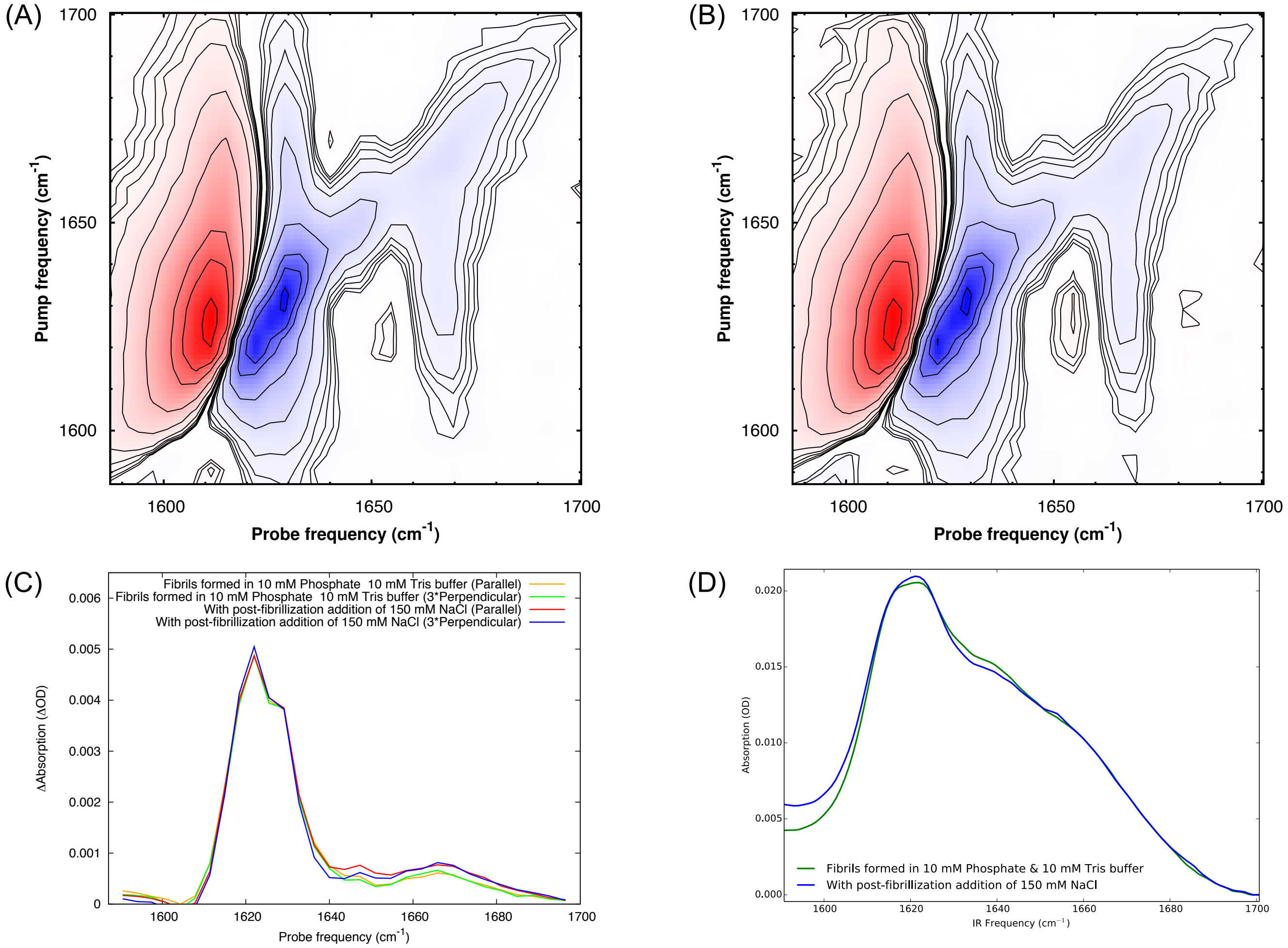

Figure 10. 2D-IR spectra of αS fibrils aggregated in the presence of only 10 mM phosphate and 10 mM Tris (A), after which 150 mM NaCl was added to the fibril solution (B), with the corresponding diagonal slices (C) and FTIR spectra (D). The contour lines are (like in all 2DIR spectra in this article) positioned at the 60th, 48th, 32th, 24th, 16th, 8th, 4th, 2nd, 1.5th and 1.1th division of the maximum and minimum.
